# Risk factors associated with pleural effusion in pediatric patients with severe Mycoplasma pneumonia

**DOI:** 10.1097/MD.0000000000050469

**Published:** 2026-09-25

**Authors:** Yanmei Teng, Hongyan Wei, Peiwen Wang, Yanfang Feng, Peipei Tian, Lingyu Li, Shuai Wang

**Affiliations:** a Pediatric Intensive Care Unit, Yihe Branch of Cangzhou People’s Hospital, Cangzhou, Hebei, China; b Pediatric Outpatient Department, Yihe Branch of Cangzhou People’s Hospital, Cangzhou, Hebei, China.

**Keywords:** C-reactive protein, fever duration, *Mycoplasma pneumoniae*, pediatric, pleural effusion, respiratory rate, risk factors, severe *Mycoplasma pneumoniae* pneumonia

## Abstract

*Mycoplasma pneumoniae* is an important cause of community-acquired pneumonia in children, and severe *M pneumoniae* pneumonia (SMPP) is frequently complicated by pleural effusion, yet specific risk factors for effusion in this population remain incompletely defined. In this retrospective observational study, 121 hospitalized children with SMPP admitted between January 2022 and June 2023 were enrolled and classified into a pleural effusion group (n = 32) and a non–pleural effusion group (n = 89) based on chest radiography. Demographic data, past medical history, clinical manifestations, laboratory indices, and radiologic findings were extracted from electronic records. Group comparisons were performed using independent-samples *t* tests and *χ*^2^ tests, and factors associated with pleural effusion were evaluated by univariate and multivariate logistic regression. Children with pleural effusion exhibited longer fever duration, higher peak temperature, faster respiratory rate, more frequent chest indrawing, and more extensive radiologic involvement than those without effusion. They also had higher white blood cell counts, neutrophil predominance, lower lymphocyte percentages, and significantly elevated C-reactive protein, procalcitonin, erythrocyte sedimentation rate, lactate dehydrogenase, and D-dimer, together with slightly lower serum albumin. Multivariate analysis identified prolonged fever duration, increased respiratory rate, and elevated C-reactive protein as independently associated with pleural effusion in children with SMPP. These readily available clinical indicators may help clinicians identify patients at higher risk of pleural effusion and facilitate closer monitoring and timely imaging evaluation.

## 1. Introduction

*Mycoplasma pneumoniae* is a common etiologic agent of community-acquired pneumonia in children, accounting for approximately 10%–40% of cases, particularly among school-aged children and adolescents.^[1,2]^ Although many infections are self-limited, *M. pneumoniae* can cause lower respiratory tract disease ranging from mild pneumonia to severe Mycoplasma pneumoniae pneumonia (SMPP) requiring hospitalization and intensive respiratory support.^[3]^ Recent epidemiologic reports and guideline updates have highlighted cyclical resurgences of *M. pneumoniae* infection and stressed the need for improved risk stratification in pediatric patients with *M pneumoniae* pneumonia ^[4,5]^ SMPP is characterized by persistent high fever, progressive radiologic consolidation, and varying degrees of hypoxemia, and may be driven by a combination of pathogen factors, host immune dysregulation, and, in some regions, macrolide resistance.^[6]^ Children with SMPP have an increased risk of pulmonary and extrapulmonary complications, including respiratory failure, necrotizing pneumonia, and involvement of the cardiovascular and nervous systems.^[7]^ Early identification of SMPP and its major complications is therefore a key component of contemporary pediatric, community-acquired pneumonia management strategies and has been incorporated into recent evidence-based recommendations.

Pleural effusion is a recognized complication of M pneumoniae pneumonia (MPP) and is frequently observed in more severe phenotypes. Population-based and hospital-based studies have reported pleural effusion in approximately 5%–26% of pediatric MPP cases, with higher frequencies among those with extensive consolidation or SMPP.^[8]^ Several recent cohorts have shown that children with MPP and pleural effusion exhibit longer fever duration, higher systemic inflammatory markers, and more extensive radiologic involvement than those without effusion, suggesting that pleural effusion is a marker of increased disease burden and a more complicated clinical course.^[9]^ Lung ultrasound and computed tomography (CT)-based assessments further indicate that even small effusions may be associated with higher lesion scores, multilobar consolidation, and the need for closer monitoring.^[10]^ Despite these observations, most recent work has concentrated on global severity outcomes such as SMPP or refractory MPP, or on prediction models for multilobar consolidation and lobar pneumonia, rather than on pleural effusion as a specific endpoint.^[11]^ Data focusing on clinical, laboratory, and imaging correlates of pleural effusion in children already classified as having SMPP remain limited. In particular, the extent to which readily available bedside variables such as fever duration, respiratory rate, and routine inflammatory indices contribute independently to pleural effusion risk in SMPP has not been clearly defined. Clarifying these relationships may help refine risk stratification, optimize the timing of imaging, and support early decisions regarding intensified monitoring and intervention.

Against this background, the present study examined a cohort of hospitalized children with SMPP to characterize the differences in clinical presentation, laboratory parameters, and radiologic findings between those with and without pleural effusion, and to identify independent risk factors associated with effusion formation using multivariate logistic regression analysis.

## 2. Methods

### 2.1. Study design

This study was approved by the Ethics Committee of Cangzhou People’s Hospital. This retrospective observational study included pediatric patients with SMPP who were hospitalized at our institution, a tertiary referral medical center, between January 2022 and June 2023. Because our hospital mainly receives children requiring hospitalization for moderate-to-severe disease, the study population may represent a subgroup with a relatively higher disease severity spectrum. According to the presence or absence of pleural fluid on chest radiography at admission or during hospitalization, patients were classified into a pleural effusion group and a non–pleural effusion group. Inclusion criteria were as follows: age between 1 month and 18 years; diagnosis of severe MPP based on clinical manifestations, radiographic findings, and microbiological evidence of *M pneumoniae* infection. *M pneumoniae* infection was confirmed based on positive *M pneumoniae*-specific IgM detected by immunoassay and/or positive nucleic acid amplification testing from respiratory specimens. Coinfection with major respiratory pathogens was excluded based on multiplex respiratory pathogen testing, bacterial culture results, and clinical evaluation. Patients with confirmed infection by other major respiratory pathogens, including influenza virus, respiratory syncytial virus, adenovirus, SARS-CoV-2, and common bacterial pathogens, were excluded; first hospitalization for the current episode; and availability of complete clinical, laboratory, and imaging data. Exclusion criteria were: congenital heart disease, congenital pulmonary malformations, or other structural cardiopulmonary abnormalities; known chronic lung disease (such as bronchopulmonary dysplasia), primary or secondary immunodeficiency, hematologic disease, or malignancy; documented co-infection with other major respiratory pathogens that could independently contribute to pleural effusion; hospital-acquired pneumonia; and incomplete or missing key medical records. The study was conducted in accordance with the principles of the Declaration of Helsinki and was approved by the institutional medical ethics committee. Written informed consent was obtained from the parents or legal guardians of all enrolled children prior to data collection.

### 2.2. Diagnostic criteria for SMPP

In this study, SMPP severity classification was determined by clinical severity indicators, including hypoxemia, extensive pulmonary involvement, persistent fever despite appropriate antimicrobial therapy, respiratory distress, extrapulmonary complications, and impaired oral intake. Pleural effusion was not considered an independent criterion for SMPP inclusion and was subsequently evaluated as the primary outcome of interest. SMPP was defined in accordance with the Chinese Guideline for the Diagnosis and Treatment of Community-Acquired Pneumonia in Children (2019), that is,as *M pneumoniae* pneumonia fulfilling at least one of the following criteria: radiological evidence of extensive pulmonary involvement, including consolidation involving ≥two-thirds of a lung or a lobe, multilobar lesions, or the presence of complications such as pleural effusion, pneumothorax, atelectasis, lung necrosis, or lung abscess; clinical and/or laboratory signs of hypoxemia, such as cyanosis, markedly increased respiratory rate with pronounced chest wall retractions, tracheal tugging or nasal flaring, and/or peripheral oxygen saturation <92% on room air; extrapulmonary organ involvement attributable to *M pneumoniae* infection (e.g., cardiovascular, neurologic, or gastrointestinal complications); persistent high fever for >5 days despite appropriate antimicrobial therapy; or significantly reduced oral intake or inability to feed.^[12]^

*M pneumoniae* infection was confirmed based on positive *M pneumoniae*-specific IgM detected by immunoassay and/or positive nucleic acid amplification testing from respiratory specimens. Coinfection with major respiratory pathogens was excluded based on multiplex respiratory pathogen testing, bacterial culture results, and clinical evaluation. Patients with confirmed infection by other major respiratory pathogens, including influenza virus, respiratory syncytial virus, adenovirus, SARS-CoV-2, and common bacterial pathogens, were excluded.

### 2.3. Data collection

Clinical data were obtained retrospectively from the electronic medical records and picture archiving and communication system of our hospital. All children who met the diagnostic criteria for severe MPP and were hospitalized between January 2022 and June 2023 were screened. For each eligible patient, a standardized data extraction form was used to collect demographic information, medical history, clinical manifestations, laboratory findings, and radiologic characteristics. Demographic and background variables included age, sex, body mass index (BMI), and relevant past medical history, such as physician-diagnosed asthma and documented allergic disease (e.g., allergic rhinitis, eczema, or food/drug allergy). Clinical data at admission comprised onset-to-admission interval (days of fever before hospitalization), peak body temperature recorded during the acute phase, respiratory rate, and the presence of chest indrawing, wheezing, and crackles on auscultation, as documented by the attending pediatrician.

Laboratory parameters were extracted from the first set of tests performed within 24 hours of admission and included complete blood count (white blood cell [WBC] count, neutrophil percentage, lymphocyte percentage, hemoglobin and platelet count], inflammatory markers (C-reactive protein [CRP], procalcitonin [PCT], erythrocyte sedimentation rate [ESR]), tissue injury and coagulation-related indices (lactate dehydrogenase [LDH], D-dimer), liver and renal function indicators [alanine aminotransferase, aspartate aminotransferase (AST), serum creatinine, blood urea nitrogen], serum albumin (ALB), electrolytes (serum sodium and potassium), and *M pneumoniae*–specific immunoglobulin M (MP-IgM) titers. Radiologic data were obtained from chest radiography and/or chest CT performed at admission or within 48 hours of admission. The following radiologic features were recorded based on official radiology reports: unilateral versus bilateral lung involvement, number of affected lobes, estimated extent of parenchymal lesions (involving <2/3 or ≥2/3 of a lung), presence of segmental or lobar consolidation, atelectasis, interstitial changes, cavitation or necrosis, and the presence or absence of pleural effusion. (Pleural effusion was identified using chest ultrasonography and/or chest CT. Chest radiography and CT performed at admission or within 48 hours were used for evaluation of pulmonary involvement. When CT was available, CT findings were prioritized because of its higher sensitivity for detecting pleural fluid and parenchymal lesions.) When both radiography and CT were available, CT findings were prioritized for classification. All data were independently extracted by 2 investigators and cross-checked for accuracy; discrepancies were resolved by discussion or consultation with a senior investigator.

Pleural effusion was defined as the presence of pleural fluid detected by chest ultrasonography and/or CT performed at admission or during hospitalization. Because imaging examinations were performed according to clinical indications during hospitalization, pleural effusion identified after admission was considered a clinical outcome associated with early disease characteristics rather than a strictly prospective event.

### 2.4. Statistical analysis

All statistical analyses were performed using IBM SPSS Statistics, version 26.0 (IBM Corp, Armonk). The distribution of continuous variables was assessed using the Shapiro–Wilk test and visual inspection of histograms and Q-Q plots. Since this is a retrospective study, all patients whose relevant information was incomplete have been excluded. Therefore, there is no data loss in this study. Continuous variables were expressed as mean ± standard deviation and categorical variables were presented as counts and percentages (n [%]). Baseline characteristics and laboratory and radiologic parameters were compared between the pleural effusion and non–pleural effusion groups using the independent-samples *t*-test for continuous variables and the *χ*^2^ test for categorical variables. Univariate logistic regression analysis was conducted with pleural effusion (yes/no) as the dependent variable to evaluate the association between each clinical, laboratory, and radiologic variable and the occurrence of pleural effusion. The results were expressed as odds ratios (ORs) with corresponding 95% confidence intervals (CIs). In the multivariate model, adjusted ORs and 95% CIs were calculated. All statistical tests were 2-sided, and a *P*-value < .05 was considered statistically significant.

Candidate variables for multivariable logistic regression were selected based on a combination of clinical relevance, biological plausibility, previous literature, and findings from univariate analyses. Considering the limited number of outcome events, the number of variables included in the final model was restricted to minimize potential overfitting. Variables representing highly correlated clinical or laboratory domains were not simultaneously entered into the model. Collinearity was assessed using variance inflation factors (VIFs), and variables with VIF values >5 were considered indicative of substantial collinearity. Multicollinearity was assessed using variance inflation factors (VIFs), and the results are presented in Supplementary Table S1, Supplemental Digital Content 1.

## 3. Results

### 3.1. General characteristics of the study population

A total of 121 children with severe MPP were included in the final analysis. The mean age of the cohort was 6.1 ± 2.2 years, and 64 (52.9%) were boys. Among these patients, 32 (26.4%) developed pleural effusion and were assigned to the pleural effusion group, while 89 (73.6%) without pleural effusion were assigned to the non–pleural effusion group. Baseline demographic and general clinical characteristics of the 2 groups are summarized in Table 1. There were no statistically significant differences between the pleural effusion and non–pleural effusion groups in age (6.2 ± 2.4 vs 6.1 ± 2.2 years, *t* = 0.22, *P* = .830), sex distribution (male: 59.4% vs 50.6%, *χ*^2^ = 0.73, *P* = .392), or BMI (16.3 ± 1.8 vs 16.5 ± 1.9 kg/m^2^, *t* = −0.52, *P* = .606). Similarly, the proportions of children with a history of asthma (21.9% vs 30.3%, *χ*^2^ = 0.83, *P* = .361) or allergic disease (25.0% vs 11.2%, *χ*^2^ = 3.52, *P* = .061) were comparable between the 2 groups. By contrast, several clinical and radiologic indicators of disease severity differed significantly according to pleural effusion status. Children in the pleural effusion group had a longer duration of fever before admission compared with those in the non–pleural effusion group (7.9 ± 2.1 vs 6.0 ± 1.7 days, t = 5.09, *P* < .001) and a higher peak body temperature (39.3 ± 0.4 vs 39.0 ± 0.4°C, *t* = 3.64, *P* < .001). The pleural effusion group also had a higher respiratory rate at presentation (39.2 ± 4.2 vs 34.7 ± 3.6 breaths/minutes, *t* = 5.80, *P* < .001) and a greater proportion of chest indrawing (68.8% vs 37.1%, *χ*^2^ = 9.52, *P* = .002), indicating more pronounced respiratory distress. Although the prevalence of wheezing and crackles on auscultation did not differ significantly between groups, lobar consolidation (71.9% vs 51.7%, *χ*^2^ = 3.91, *P* = .048) and atelectasis (34.4% vs 16.9%, *χ*^2^ = 4.28, *P* = .038) were significantly more frequent in the pleural effusion group (Table 1).

**Table 1:** Baseline demographic and clinical characteristics of children with severe *Mycoplasma pneumoniae* pneumonia according to pleural effusion status.

Variable	Pleural effusion group (n = 32)	Non–pleural effusion group(n = 89)	Test statistic	*p*-value
Age, yr	6.2 ± 2.4	6.1 ± 2.2	*t* = 0.22	.830
Male sex, n (%)	19 (59.4)	45 (50.6)	*χ*^2^ = 0.73	.392
BMI, kg/m^2^	16.3 ± 1.8	16.5 ± 1.9	*t* = −0.52	.606
History of asthma, n (%)	7 (21.9)	27 (30.3)	*χ*^2^ = 0.83	.361
History of allergic disease, n (%)	8 (25.0)	10 (11.2)	*χ*^2^ = 3.52	.061
Fever duration, days	7.9 ± 2.1	6.0 ± 1.7	*t* = 5.09	<.001
Peak temperature, °C	39.3 ± 0.4	39.0 ± 0.4	*t* = 3.64	<.001
Wheezing, n (%)	16 (50.0)	46 (51.7)	*χ*^2^ = 0.03	.870
Respiratory rate, breaths/min	39.2 ± 4.2	34.7 ± 3.6	*t* = 5.80	<.001
Chest indrawing, n (%)	22 (68.8)	33 (37.1)	*χ*^2^ = 9.52	.002
Crackles on auscultation, n (%)	28 (87.5)	67 (75.3)	*χ*^2^ = 2.08	.149
Lobar consolidation, n (%)	23 (71.9)	46 (51.7)	*χ*^2^ = 3.91	.048
Atelectasis, n (%)	11 (34.4)	15 (16.9)	*χ*^2^ = 4.28	.038

BMI = body mass index, SD = standard deviation.

### 3.2. Comparison of laboratory and radiologic characteristics between groups

WBC counts were significantly higher in the pleural effusion group than in the non–pleural effusion group (13.2 ± 4.1 vs 10.7 ± 3.6 × 10^9^/L; *t* = 3.05, *P* = .004). The neutrophil percentage was increased (78.5 ± 8.2% vs. 71.3 ± 9.4%; t = 4.09, *P* < .001), whereas the lymphocyte percentage was reduced (15.6 ± 6.4% vs 20.3 ± 7.2%; *t* = −3.44, *P* = .001) in the pleural effusion group. In contrast, hemoglobin concentration and platelet counts did not differ significantly between groups (both *P* > .05). Inflammatory biomarkers were consistently higher in the pleural effusion group. CRP (76.4 ± 32.5 vs 49.2 ± 27.8 mg/L; *t* = 4.21, *P* < .001), PCT (1.12 ± 0.85 vs 0.54 ± 0.49 ng/mL; *t* = 3.65, *P* < .001), ESR (43.5 ± 16.4 vs 31.2 ± 13.7 mm/hours; *t* = 3.79, *P* < .001), LDH (498 ± 151 vs 402 ± 132 U/L; *t* = 3.19, *P* = .003), and D-dimer (1.52 ± 0.98 vs 0.89 ± 0.63 mg/L; *t* = 3.39, *P* = .002) were all significantly elevated in children with pleural effusion. Liver and renal function indices (alanine aminotransferase, AST, creatinine, and blood urea nitrogen) and electrolytes (serum sodium and potassium) showed no statistically significant between-group differences (all *P* > .05). Serum ALB was modestly but significantly lower in the pleural effusion group (37.9 ± 4.1 vs 39.8 ± 4.3 g/L; *t* =−2.22, *P* = .030). *M pneumoniae*–specific immunoglobulin M (MP-IgM) titers tended to be higher in the pleural effusion group, with borderline statistical significance (1.78 ± 0.65 vs 1.52 ± 0.61 arbitrary units; *t* = 1.97, *P* = .054). Radiologic findings indicated more extensive pulmonary involvement in the pleural effusion group. Bilateral lung involvement was more frequent in children with pleural effusion than in those without (81.3% vs 48.3%; *χ*^2^ = 10.42, *P* = .001). Similarly, involvement of 2 or more lobes (75.0% vs 42.7%; *χ*^2^ = 9.83, *P* = .002) and lesions affecting at least two-thirds of a lung (43.8% vs 19.1%; *χ*^2^ = 7.50, *P* = .006) were significantly more common in the pleural effusion group. Segmental or lobar consolidation (71.9% vs 51.7%; *χ*^2^ = 3.91, *P* = .048), atelectasis (34.4% vs 16.9%; *χ*^2^ = 4.28, *P* = .039), and interstitial changes (56.3% vs 34.8%; *χ*^2^ = 4.48, *P* = .034) also showed higher frequencies in the pleural effusion group. Cavitation or necrotic changes remained uncommon in both groups and did not differ significantly (9.4% vs 2.2%; *χ*^2^ = 3.02, *P* = .082) (Table 2).

**Table 2:** Comparison of laboratory and radiologic characteristics between children with and without pleural effusion.

Variable	Pleural effusion group (n = 32)	Non–pleural effusion group (n = 89)	Test statistic	*P*-value
Laboratory parameters				
WBC, ×10^9^/L	13.2 ± 4.1	10.7 ± 3.6	*t* = 3.05	.004
Neutrophils, %	78.5 ± 8.2	71.3 ± 9.4	*t* = 4.09	<.001
Lymphocytes, %	15.6 ± 6.4	20.3 ± 7.2	*t* = −3.44	.001
Hb, g/L	115.0 ± 12.0	118.0 ± 11.0	*t* = −1.24	.221
PLT, ×10^9^/L	348 ± 92	321 ± 85	*t* = 1.45	.153
CRP, mg/L	76.4 ± 32.5	49.2 ± 27.8	*t* = 4.21	<.001
PCT, ng/mL	1.12 ± 0.85	0.54 ± 0.49	*t* = 3.65	<.001
ESR, mm/h	43.5 ± 16.4	31.2 ± 13.7	*t* = 3.79	<.001
LDH, U/L	498 ± 151	402 ± 132	*t* = 3.19	.003
D-dimer, mg/L	1.52 ± 0.98	0.89 ± 0.63	*t* = 3.39	.002
ALT, U/L	38.6 ± 18.7	34.1 ± 16.8	*t* = 1.20	.236
AST, U/L	44.7 ± 19.2	37.5 ± 17.3	*t* = 1.87	.068
Cr, μmol/L	41.2 ± 9.8	39.5 ± 8.9	*t* = 0.86	.393
BUN, mmol/L	4.8 ± 1.2	4.5 ± 1.1	*t* = 1.24	0.221
ALB, g/L	37.9 ± 4.1	39.8 ± 4.3	*t* = −2.22	.030
Sodium, mmol/L	135.6 ± 3.4	136.7 ± 3.2	*t* = −1.59	.117
Potassium, mmol/L	3.8 ± 0.4	3.9 ± 0.4	*t* = −1.21	.230
MP-IgM, arbitrary units	1.78 ± 0.65	1.52 ± 0.61	*t* = 1.97	.054
Radiologic characteristics				
Bilateral lung involvement, n (%)	26 (81.3)	43 (48.3)	*χ*^2^ = 10.42	.001
Involvement of ≥2 lobes, n (%)	24 (75.0)	38 (42.7)	*χ*^2^ = 9.83	.002
Lesions involving ≥2/3 of a lung, n (%)	14 (43.8)	17 (19.1)	*χ*^2^ = 7.50	.006
Segmental/lobar consolidation, n (%)	23 (71.9)	46 (51.7)	*χ*^2^ = 3.91	.048
Atelectasis, n (%)	11 (34.4)	15 (16.9)	*χ*^2^ = 4.28	.039
Interstitial changes, n (%)	18 (56.3)	31 (34.8)	*χ*^2^ = 4.48	.034
Cavitation/necrosis, n (%)	3 (9.4)	2 (2.2)	*χ*^2^ = 3.02	.082

ALB = albumin, ALT = alanine aminotransferase, AST = aspartate aminotransferase, BUN = blood urea nitrogen, Cr = creatinine, CRP = C-reactive protein, ESR = erythrocyte sedimentation rate, Hb = hemoglobin, LDH = lactate dehydrogenase, MP-IgM = *Mycoplasma pneumoniae*–specific immunoglobulin M, PCT = procalcitonin, PLT = platelet count, SD = standard deviation, WBC = white blood cell count.

### 3.3. Univariate logistic regression analysis of factors associated with pleural effusion

Using pleural effusion (yes = 1, no = 0) as the dependent variable, univariate logistic regression was performed for demographic, clinical, laboratory, and radiologic variables. Among demographic and clinical variables, age, sex, BMI, and history of asthma were not significantly associated with pleural effusion (all *P* > .10). History of allergic disease showed a borderline association with pleural effusion (OR = 2.63, 95% CI: 0.93–7.42, *P* = .067). Fever duration, peak temperature, respiratory rate, and chest indrawing were all significantly associated with the presence of pleural effusion (all *P* < .01). For laboratory indices, higher WBC, higher neutrophil percentage, and lower lymphocyte percentage were significantly associated with pleural effusion (all *P* ≤ .01). CRP, PCT, ESR, and D-dimer were significantly associated with pleural effusion (all *P* ≤ .022). Lower ALB and higher AST were also significantly associated with pleural effusion (both *P* < .01). LDH and *M pneumoniae*–specific immunoglobulin M were not significantly associated with pleural effusion (both *P* > .10). For radiologic characteristics, bilateral lung involvement, involvement of ≥2 lobes, and lesions involving ≥2/3 of a lung were significantly associated with pleural effusion (all *P* < .01). Atelectasis and interstitial changes were also significantly associated with pleural effusion (*P* < .05). Segmental/lobar consolidation showed borderline significance (*P* = .051), and cavitation/necrosis was not significantly associated with pleural effusion (Table 3).

**Table 3:** Univariate logistic regression analysis of factors associated with pleural effusion in children with severe *Mycoplasma pneumoniae* pneumonia.

Variable	OR	95% CI	*P*-value
Age, yr	0.97	0.80–1.18	.774
Male sex	1.43	0.63–3.24	.393
BMI, kg/m^2^	0.83	0.65–1.04	.103
History of asthma	0.64	0.25–1.67	.363
History of allergic disease	2.63	0.93–7.42	.067
Fever duration, days	1.66	1.28–2.15	<.001
Peak temperature, °C	8.41	2.83–24.99	<.001
Respiratory rate, breaths/min	1.40	1.20–1.63	<.001
Chest indrawing	3.73	1.58–8.84	.003
WBC, ×10^9^/L	1.24	1.11–1.39	<.001
Neutrophils, %	1.07	1.02–1.13	.007
Lymphocytes, %	0.87	0.81–0.94	<.001
CRP, mg/L	1.02	1.00–1.03	.022
PCT, ng/mL	10.78	4.06–28.59	<.001
ESR, mm/h	1.08	1.04–1.12	<.001
LDH, U/L	1.00	1.00–1.01	.168
D-dimer, mg/L	3.67	1.94–6.97	<.001
ALB, g/L	0.80	0.71–0.90	<.001
AST, U/L	1.04	1.01–1.07	.002
MP-IgM, arbitrary units	1.37	0.70–2.68	.353
Bilateral lung involvement	4.64	1.74–12.35	.002
Involvement of ≥2 lobes	4.03	1.63–9.94	.003
Lesions involving ≥2/3 of a lung	3.29	1.37–7.91	.008
Segmental/lobar consolidation	2.39	1.00–5.73	.051
Atelectasis	2.58	1.03–6.46	.042
Interstitial changes	2.41	1.06–5.48	.037
Cavitation/necrosis	4.50	0.72–28.28	.109

ALB = albumin, AST = aspartate aminotransferase, BMI = body mass index, CI = confidence interval, CRP = C-reactive protein, ESR = erythrocyte sedimentation rate, LDH = lactate dehydrogenase, MP-IgM = *Mycoplasma pneumoniae*–specific immunoglobulin M, OR = odds ratio, PCT = procalcitonin, WBC = white blood cell count.

### 3.4. Multivariate logistic regression analysis of risk factors for pleural effusion

Considering the limited number of pleural effusion events (n = 32), only clinically relevant variables supported by previous literature and showing potential associations in univariate analyses were considered for multivariable modeling. After evaluation of collinearity and clinical interpretability, 5 variables were included in the final logistic regression model: fever duration, respiratory rate, CRP level, serum ALB, and bilateral lung involvement. The results are shown in Table 4. Multivariate analysis identified fever duration, respiratory rate, and CRP as independent risk factors for pleural effusion in children with . Longer fever duration (OR = 1.96, 95% CI: 1.40–2.73, *P* < .001), higher respiratory rate (OR = 1.45, 95% CI: 1.16–1.80, *P* = .001), and higher CRP level (OR = 1.03, 95% CI: 1.00–1.05, *P* = .031) were independently associated with increased odds of pleural effusion. Serum ALB and bilateral lung involvement did not remain statistically significant after adjustment for these factors (*P* = .330 and *P* = .143, respectively) (Table 4). No substantial collinearity was detected among variables included in the final model (all VIFs < 2.0; Supplementary Table S1, Supplemental Digital Content 1).

**Table 4:** Multivariable logistic regression analysis of factors associated with pleural effusion.

Variable	β	SE	OR	95% CI for OR	*P*-value
Fever duration, d	0.67	0.17	1.96	1.40–2.73	<.001
Respiratory rate, breaths/min	0.37	0.11	1.45	1.16–1.80	.001
CRP, mg/L	0.03	0.01	1.03	1.00–1.05	.031
ALB, g/L	−0.09	0.09	0.92	0.77–1.09	.330
Bilateral lung involvement (yes vs no)	1.03	0.70	2.79	0.71–11.05	.143

ALB = albumin, CI = confidence interval, CRP = C-reactive protein, OR = odds ratio, SE = standard error, β = regression coefficient.

## 4. Discussion

This study systematically evaluated clinical, laboratory, and radiologic factors associated with pleural effusion in children with (SMPP). Approximately 1-quarter of the cohort (26.4%) developed pleural effusion, and these patients exhibited more severe systemic inflammation and more extensive pulmonary involvement than those without effusion. Multivariate logistic regression identified 3 independent risk factors for pleural effusion: longer fever duration before admission, higher respiratory rate at presentation, and elevated CRP concentration. In contrast, baseline demographic variables and most routine biochemical indices were not independently related to pleural effusion. The association between prolonged fever and pleural effusion suggests that persistent systemic inflammatory activity is central to effusion formation in SMPP. Longer febrile courses likely reflect sustained host immune activation and delayed control of infection, which may promote endothelial injury, increased capillary permeability, and exudation into the pleural space. The independent effect of respiratory rate further supports the link between pleural effusion and more severe respiratory compromise. Tachypnea is an integrative marker of increased work of breathing, impaired gas exchange, and reduced pulmonary compliance; its association with pleural effusion indicates that effusion occurs in the context of more extensive or functionally important parenchymal disease.

CRP emerged as an independent laboratory predictor, whereas other inflammatory markers such as PCT, ESR, LDH, and D-dimer, although significantly higher in the pleural effusion group, did not retain significance after adjustment. CRP is a robust, nonspecific marker of systemic inflammation and tissue injury. Its independent association with pleural effusion suggests that overall inflammatory burden, rather than any single pathway, is critical for effusion development in SMPP. The loss of significance for ALB and bilateral lung involvement in the multivariate model indicates that their effects may be mediated through or confounded by fever duration, tachypnea, and CRP, which together capture both systemic and local disease severity. Hypoalbuminemia in the pleural effusion group, although not an independent predictor, is still clinically relevant. Lower ALB levels may at least partly reflect capillary leak, inflammatory consumption, and reduced nutritional reserve, thereby lowering plasma oncotic pressure and favoring fluid transudation into the pleural cavity. Similarly, the higher frequency of bilateral, multilobar, and extensive radiologic lesions among children with pleural effusion is consistent with the notion that effusion is a marker of more widespread lung involvement. These patterns support a model in which extensive parenchymal inflammation, sustained febrile response, and systemic inflammation converge to promote pleural fluid accumulation.

The proportion of SMPP patients with pleural effusion in the present study (26.4%) is comparable to previously reported rates of pleural effusion in pediatric *M pneumoniae* pneumonia, which range from approximately 15% to 25% in mixed-severity cohorts.^[13]^ Recent multicenter and single-center studies have confirmed that pleural effusion is more frequent in children with more severe disease and is associated with higher inflammatory indices and more extensive radiologic involvement.^[13,14]^ Several recent investigations have specifically focused on pleural effusion or effusion-related complications in *M pneumoniae* infection. Ji et al analyzed pediatric MPP with and without pleural effusion and found that patients with effusion had longer fever duration, higher CRP and LDH levels, and more extensive lung involvement; prolonged fever and elevated CRP were independent predictors of effusion, broadly consistent with the present findings.^[15]^ Kim et al reported that MPP patients with pleural effusion had more severe clinical courses and higher inflammatory markers than those without effusion, again highlighting pleural effusion as a marker of heightened inflammatory response.^[13]^ In children with pneumonia-related plastic bronchitis, Lin et al showed that the combined detection of CRP and LDH improved prediction of pleural effusion, supporting the role of systemic inflammation and tissue injury markers in identifying patients at risk of effusion.^[16]^ More broadly, Wang et al demonstrated that pleural effusion itself was a significant risk factor for complications in MPP, emphasizing its value as a clinical warning sign in the disease course.^[17]^

Although many recent studies have focused on refractory MPP, refractory Mycoplasma pneumoniae pneumonia (RMPP) or SMPP rather than pleural effusion per se, their observations are highly concordant with the present work. Multiple predictive models for RMPP or SMPP have repeatedly identified prolonged fever duration, elevated CRP, ESR, and LDH, and extensive radiologic involvement as major risk factors for severe outcomes.^[18,19]^ Li et al developed a nomogram for RMPP and found that fever duration, CRP, and LDH were among the most informative predictors^[20]^; Shen et al and subsequent studies similarly identified CRP and fever duration as key early indicators of refractory disease. A recent dynamic nomogram for predicting SMPP risk also incorporated fever days ≥7 and CRP as independent predictors, along with pleural effusion itself as a marker of severe disease. These data collectively support the central role of sustained fever and systemic inflammation in driving severe pulmonary manifestations, including pleural effusion, in pediatric MPP. The present study adds to this literature by focusing specifically on SMPP and quantifying the independent contributions of fever duration, respiratory rate, and CRP to pleural effusion risk, while controlling for extensive radiologic involvement and hypoalbuminemia. This targeted perspective complements existing RMPP and SMPP prediction models by clarifying which routinely available bedside and laboratory variables are most informative for identifying SMPP patients at increased risk of effusion.

Several limitations should also be acknowledged. First, this was a retrospective, single-center study, and selection bias cannot be completely excluded. As our institution is a tertiary referral center, hospitalized patients may represent children with more severe clinical manifestations compared with the general population of children with MPP. In addition, variations in antimicrobial treatment timing, corticosteroid administration, immunomodulatory therapy, and supportive management may have influenced the development or recognition of pleural effusion. Although we adjusted for several clinically relevant factors, residual confounding remains possible. Second, although pleural effusion was identified using chest ultrasonography and/or CT, detailed classification according to effusion volume, loculation, and pleural fluid biochemical characteristics was unavailable because pleural fluid analysis was not routinely performed in all patients. Therefore, we were unable to determine whether the identified associated factors differed between small, uncomplicated effusions and more clinically significant, complicated effusions requiring intervention. Future prospective studies incorporating standardized assessment of pleural effusion characteristics are warranted. Finally, the study did not assess long-term respiratory outcomes, so the prognostic implications of pleural effusion beyond acute hospitalization remain uncertain.

## 5. Conclusion

In children with SMPP, pleural effusion was associated with more marked systemic inflammation and more extensive pulmonary involvement. Multivariate analysis identified prolonged fever duration, increased respiratory rate, and elevated CRP as independent risk factors for pleural effusion. These readily available clinical and laboratory parameters may facilitate early risk stratification and support timely diagnostic and therapeutic decision-making.

## Author contributions

**Conceptualization:** Yanmei Teng, Hongyan Wei, Peiwen Wang, Yanfang Feng, Peipei Tian, Lingyu Li, Shuai Wang.

**Data curation:** Yanmei Teng, Hongyan Wei, Peiwen Wang, Yanfang Feng, Peipei Tian, Lingyu Li, Shuai Wang.

**Formal analysis:** Yanmei Teng, Hongyan Wei, Peiwen Wang, Yanfang Feng, Peipei Tian, Lingyu Li, Shuai Wang.

**Funding acquisition:** Yanmei Teng, Shuai Wang.

**Investigation:** Shuai Wang.

**Writing – original draft:** Yanmei Teng, Shuai Wang.

**Writing – review & editing:** Yanmei Teng, Shuai Wang.


