# Supplementary material for: Risk factors associated with pleural effusion in pediatric patients with severe Mycoplasma pneumonia
Source: Medicine (Baltimore). 2026 Sep 25;105(39):e50469. doi: 10.1097/MD.0000000000050469 (PMC13619238; doi:10.1097/MD.0000000000050469)
Supplement: Supplementary file 1 [file medi-105-e50469-s001.docx]

**Supplementary Table S1. Assessment of multicollinearity among variables included in the multivariable logistic regression model.**

| **Variable** | **Variance inflation factor (VIF)** | **Tolerance** |
| --- | --- | --- |
| Fever duration | 1.42 | 0.70 |
| Respiratory rate | 1.76 | 0.57 |
| C-reactive protein (CRP) | 1.83 | 0.55 |
| Serum albumin | 1.31 | 0.76 |
| Bilateral lung involvement | 1.56 | 0.64 |

Note: VIF, variance inflation factor.
